# Biochemical and antigenic characterization of the structural proteins and their post-translational modifications in purified SARS-CoV-2 virions of an inactivated vaccine candidate

**DOI:** 10.1080/22221751.2020.1855945

**Published:** 2020-12-14

**Authors:** Xiao-Yu Zhang, Jing Guo, Xin Wan, Jin-Ge Zhou, Wei-Ping Jin, Jia Lu, Wen-Hui Wang, An-Na Yang, Ding Xiang Liu, Zheng-Li Shi, Zhi-Ming Yuan, Xin-Guo Li, Sheng-Li Meng, Kai Duan, Ze-Jun Wang, Xiao-Ming Yang, Shuo Shen

**Affiliations:** a Wuhan Institute of Biological Products, Co. Ltd, Wuhan, People’s Republic of China; b South China Agricultural University, Guangdong Province Key Laboratory Microbial Signals & Disease Co, & Integrative Microbiology Research Center, Guangzhou, People’s Republic of China; c Wuhan Institute of Virology, Center for Biosafety Mega-Science, Chinese Academy of Sciences, Wuhan, People’s Republic of China; d China National Biotech Group Company Ltd, Beijing, People’s Republic of China

**Keywords:** Inactivated vaccine, SARS-CoV-2, structural proteins, modifications, antigenicity

## Abstract

In the face of COVID-19 pandemic caused by the newly emerged SARS-CoV-2, an inactivated, Vero cell-based, whole virion vaccine candidate has been developed and entered into phase III clinical trials within six months. Biochemical and immunogenic characterization of structural proteins and their post-translational modifications in virions, the end-products of the vaccine candidate, would be essential for the quality control and process development of vaccine products and for studying the immunogenicity and pathogenesis of SARS-CoV-2. By using a panel of rabbit antisera against virions and five structural proteins together with a convalescent serum, the spike (S) glycoprotein was shown to be N-linked glycosylated, PNGase F-sensitive, endoglycosidase H-resistant and cleaved by Furin-like proteases into S1 and S2 subunits. The full-length S and S1/S2 subunits could form homodimers/trimers. The membrane (M) protein was partially N-linked glycosylated; the accessory protein 3a existed in three different forms, indicative of cleavage and dimerization. Furthermore, analysis of the antigenicity of these proteins and their post-translationally modified forms demonstrated that S protein induced the strongest antibody response in both convalescent and immunized animal sera. Interestingly, immunization with the inactivated vaccine did not elicit antibody response against the S2 subunit, whereas strong antibody response against both S1 and S2 subunits was detected in the convalescent serum. Moreover, vaccination stimulated stronger antibody response against S multimers than did the natural infection. This study revealed that the native S glycoprotein stimulated neutralizing antibodies, while bacterially-expressed S fragments did not. The study on S modifications would facilitate design of S-based anti-SARS-CoV-2 vaccines.

## Introduction

At the end of 2019, a novel, zoonotic coronavirus jumped across the species barrier to transmit to human, soon causing pandemic and impacting unprecedently on life, health care, social life of humanity and on global economy. The coronavirus disease 2019 (COVID-19) is caused by severe acute respiratory syndrome coronavirus-2 (SARS-CoV-2) [1–3] and characterized with fever, severe respiratory illness and pneumonia. Development of vaccines against COVID-19 is critical to limit further damages. Biochemical characterization on the newly emerged SARS-CoV-2 is important for better understanding of pathogenesis and immunology and may benefit development of therapeutics and vaccines.

SARS-CoV-2 belongs the family *Coronaviridae*, subfamily *Coronavirinae,* genera *Betacoronavirus*. Within genera *Betacoronavirus*, four lineages A, B, C and D could be phylogenetically distinguished and both SARS-CoV-1 and SARS-CoV-2 belong to the same lineage B. The two viruses share 79.6% homology in the nucleotide sequence of the genome and the same main receptor, angiotensin-converting enzyme 2 (ACE2) for binding and entry into cells [2]. The genome of SARS-CoV-2 is approximate 29 kilobases in length, the largest among all RNA viruses. The genomic RNA is flanked by 5′- and 3′ untranslated regions with 5′-cap and 3′-poly (A)_n_ tail. The 5′-two thirds of the genome contain open reading frames (ORF) encoding nonstructural polyproteins 1a and 1ab and the 3′-one third ORFs encoding structural proteins and accessory proteins. Cleavages of polyproteins of 1a/1ab and post-translational modification of a variety of structural and nonstructural proteins result in mature and functional viral proteins [4]. Morphologically, SARS-CoV-2 is spherical or pleomorphic in shape with an average diameter of 91 ± 11 nm [5].

The main structural proteins of SARS-CoV-2 are the spike glycoprotein (S), nucleocapsid protein (N), membrane glycoprotein (M), accessory 3a protein and envelop protein (E). The glycosylation, cleavage, other post-translational modification and trimerization are essential for functions of some of these proteins. The glycosylated S, M, E and multiform 3a are viral membrane proteins, while the phosphorylated N protein binds to viral genomic RNA in a beads-on-one-string fashion [4]. The M and E proteins play essential roles in particle assembling [6]. Previous reports showed that all these proteins stimulated humoral immune responses after SARS-CoV-1 infection, strongly or weakly [7]. The major neutralizing antigen is the S glycoprotein, but a monoclonal antibody against M also showed neutralizing activity and protection against virus challenge [8]. Kim et al reported that DNA vaccine targeting the N protein triggered specific humoral and T-cell-mediated immune responses [9]. However, the researches on N protein of CoV families suggested that it was not an optimal vaccine candidate [10]. S protein is cleaved by cellular Furin-like proteases into S1 and S2 subunits, which modulates receptor binding and virus-cell membrane fusion, respectively, at the steps of virus entry into cells. The folding, disulfide bond formation, N-linked glycosylation and polymerization are critical for maturation, native conformation and function of S protein. Its conformational changes have important impacts on the propagation, immunogenicity and pathogenesis of CoVs, and biochemical characterization of S protein is particularly important as it is the main target for vaccine design. 3a is an accessory protein unique to SARS-CoV-1 and SARS-CoV-2. It was a viral membrane protein [11], transported to cell membrane and Golgi apparatus[4,12–14]. 3a dimers and tetramers form ion channel regulating apoptosis [15]. Modulation of 3a ion channel activity may inhibit SARS-CoV-1 replication and infectivity [16]. The E protein is a small transmembrane protein present at a low proportion which is essential in virion assembling and releasing [17,18].

Biochemical and immunogenic characterization of the SARS-CoV-2 structural proteins is warranted for the development of therapeutics and vaccines. In this study, the viral structural proteins and their post-translational modifications were characterized using antibodies raised against whole virions, different regions of the S glycoprotein and other four structural proteins, N, M, 3a and E, and a convalescent serum. Interestingly, the N-linked glycosylated full-length S, cleaved S1 and S2 subunits were shown to form trimers/dimers and to be highly immunogenic. This finding and a deeper understanding of the structural protein profile and their post-translational modifications would be beneficial for design of safe and effective vaccine candidates. The detailed characterization of these modifications will also be of help to establish simple and quick methods for the quality control in the production of this vaccine and other S-based vaccines.

## Materials and methods

### Cells and virus

Vero cells were maintained in complete DMEM medium (Gibco), supplemented with newborn calf serum (NCS, 10%), streptomycin (0.1 mg/ml) and penicillin (100 units/ml) (Gibco). Cells were infected at a multiplicity of infection of 0.1–0.001 plaque forming unit per cell. Viruses were cultured in maintenance medium (DMEM) supplemented with 1% antibiotic-antimycotic (Gibco, 15240-062) in the absence of NCS. The isolation and the complete genome sequence of the WIV-04 strain (IVCAS 6.7512) of SARS-CoV-2 was described previously [2].

### Purification of SARS-CoV-2

The processes of the vaccine preparation and quality control methods were submitted for patent applications. Briefly, the processes included the cell culture and virus propagation, harvesting, β-propiolactone-inactivation (1:4000 (v/v)) at 2–8°C for 48 h, followed by cell debris clarification, ultrafiltration, 2nd β-propiolactone-inactivation, gel-chromatography, ion-exchange chromatography, sterile filtration, formulation with buffer and aluminum hydroxide (Alum), filling, packaging and labelling. The intermediate products before formulation with Alum was used for this study. Samples before formulation process were taken for biochemical analysis of virions.

### Preparation of antibodies and collection of convalescent sera

Convalescent sera were collected from recovered patients as previously described [19]. Cloning of genes, expressing and purification of Coronavirus proteins in *E. coli* for immunization of rabbits were described previously [20]. Briefly, rabbit antisera against individual structural proteins were raised using N-terminal GST-tagged, *E. coli* cell-expressed fragments of the S (SΔ1/2-Aa507-786 and S4Δ-Aa768-1024, SΔ5-Aa1045-1243), M (MΔ1-Aa150-222), 3a (3aΔ1-Aa157-274), and the full-length N and E proteins. Rabbit anti-RBD serum was purchased from SinoBilogical (Cat No., 40592-T62) and was referred to as rabbit α-S1(Aa319-541). Two to four hundred micrograms of each of these proteins was mixed with an equal volume of complete Freund’s adjuvant (Sigma) and used for the immunization of Japanese White rabbits. Two weeks after the priming, the rabbits were given booster injections at 2-week intervals. Incomplete Freund’s adjuvant (Sigma) was used for subsequent booster injections. Ten millilitres of blood were collected from the rabbits each time 10 or 14 days after the 4th and 6th injections. This study protocol was approved by the Animal Ethics Committee of the Wuhan Institute of Biological Products (WIBP) (WIBP-AII no.382020002). All experiments were performed in accordance with the relevant guidelines and regulations.

### SDS-PAGE and Western blotting analysis of viral structural proteins

The purified vaccine was concentrated by using Amicon Ultra-15 centrifuge tube (Ultracel-3 kDa). After centrifugation at 3000×*g* at 4°C for 3 h, the samples were subjected to poly-acrylamide gel electrophoresis (PAGE). Samples for Western blotting (WB) analysis were diluted properly and were incubated with antibodies directed against the virions and individual proteins. Viral proteins were separated on 4–20% or 4–12% gradient or 8% polyacrylamide gels (for separating S1 smear bands). The transfer of proteins to nitrocellulose filter membrane was performed at 380 mA at 4°C for 70 min.

### Endoglycosidase H (Endo H) and PNGase F digestion for deglycosylation of viral glycoproteins

Ten micrograms of viral proteins were boiled in 1 × glycoprotein denaturation buffer (containing 0.5% SDS, 40 mM DTT) at 100°C for 10 min for denature and then were incubated in 1× Glyco Buffer 3 containing 500 units Endo H (NEB) at 37°C for 1 h following manufacturer’s protocol. Thermal inactivation was performed at 65°C for 10 min. Ten micrograms of viral proteins were heated in 1 × glycoprotein denaturation buffer (containing 0.5% SDS, 40 mM DTT) at 100°C for 10 min and then incubate at 37°C in 1 × Glyco Buffer 2 containing 1% NP-40 and PNGase F (500 units). Thermal inactivation was performed at 75°C for 10 min.

### Furin digestion and of S protein

Two tubes containing 10 μg viral protein each were incubated in 20 mM HEPES containing 0.1% Triton X-100, 0.2 mM CaCl_2_, 0.2 mM β-Mercaptoethanol in a total reaction volume of 25 μl. Two units of Furin (NEB) were added to one tube. Two units Furin (NEB) and 200 uM Furin inhibitor (Abcam) were added to another tube. Both of the tubes were incubated at 25°C for 6 h.

### Detection of the S trimers/dimers

Loading buffer (4×) was used to mix for SDS-PAGE containing 50 mM Tris-HCl, pH 6.8, 2% SDS, 100 mM β-Mercaptoethanol, 10% glycerol, and 0.1% bromophenol blue. In the case of detection of the S trimers, 2% NP-40 was contained in the loading buffer. The trimer dissociation loading buffer contains 6 M Urea and 2% NP-40 and proteins were separated by 0.1% SDS-8% PAGE.

## Results

### Generation of antisera against SARS-CoV-2 structural proteins

**Figure 1.**
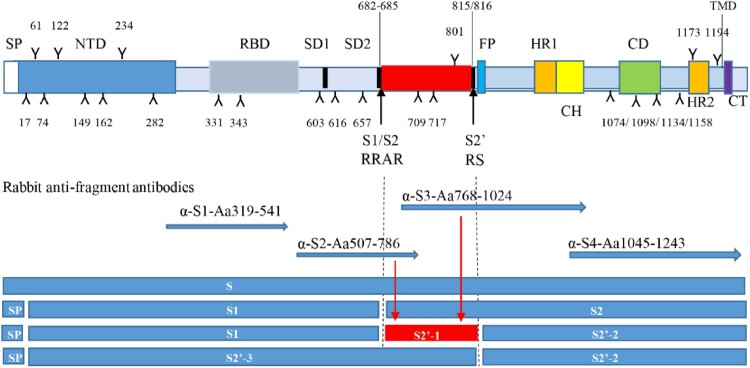
Potential cleavage products if Furin and host proteases cleaved at both S1/S2 and S2′ (not in scale). The function domains of the S are illustrated and potential N-linked glycosylation sites indicated by Y and numbers. Predicted cleavage sites of S are indicated by lines and arrowhead. Rabbit anti-S fragment antisera are prepared or purchased and their targeted regions are indicated by arrowhead lines. Potential cleavage products are showed and referred as to S1, S2, S2′-1, S2′-2 and S2′-3 if S1/S2 and S2′ cleavage sites (positions and amino acids indicated) are used. SP, signal peptide; NTD, N-terminal domain; RBD, receptor binding domain; SD1/SD2, subdomains 1 and 2; FP, fusion peptide; HR1/HR2, heptad repeats 1 and 2; CH, central helix; CD, Connector domain; TMD, transmembrane domain; CT, cytoplasmic tail.

To characterize the structural protein profiles in the virion of an inactivated and whole virus vaccine, antisera against purified virions, five major structural proteins (S, N, M, E and 3a) were raised in rabbits. Full-length or fragments of these proteins were expressed as GST-tagged fusion proteins. For characterization of the S glycoprotein, four antisera, α-S1, α-S2, α-S3, α-S4, against different regions of the protein, as shown in Figure 1, were raised. These S-specific antisera would recognize different fragments of S if the protein was cleaved by host cell proteases [21]. Rabbit anti-virions stimulates high titres of neutralizing antibodies (Submitted for publication), while the S fragments expressed in *E. coli* did not induced functional neutralizing antibodies (Data not shown). The titres of neutralizing antibodies reached to 5120, 5120, respectively, following two injections of the candidate vaccine at a dose of 25 µg. The results are consistent with our previous study on the immunogenicity of SARS-CoV-1 S protein [20].

### Viral structural protein profiles in virions of the vaccine strain

**Figure 2.**
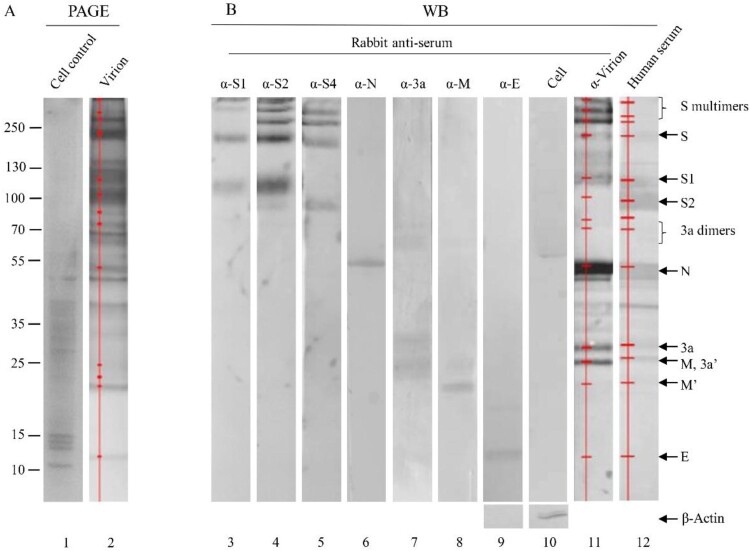
Identification of viral proteins in an inactivated, whole virion vaccine. (A) The proteins were separated by 4-20% of gradient SDS-PAGE and silver stained. (B) The separated proteins were transferred to Nitrocellulose membrane for WB using human convalescent serum and rabbit antisera against virion and individual proteins as indicated on the top. 3a′, cleaved; M′, unglycosylated. Protein profiles in PAGE (Lane 2) and WB (Lanes 11 and 12) were scanned (Red lines and dots). Mouse α-β-Actin mAb was used as loading controls. Molecular weight markers are indicated on the left in kilodaltons and proteins are indicated by the arrows on the right. The experiments were repeated 10 times at least.

To gain an overall profile of structural proteins present in the SARS-CoV-2 virions, viral proteins in inactivated, purified virions were separated on SDS-4-12% PAGE and visualized with sensitive silver-staining (Figure 2A, lane 2). The virus-specific protein components were initially identified by comparing with protein bands in the total lysates of mock-infected cells (Figure 2A, lanes 1 and 2). The identity of each viral protein was then confirmed using specific antisera against individual proteins, whole virions and a convalescent serum from a recovered patient (Figure 2B). When α-S3 was used, the protein profile was the same as that of the rabbit α-S4 (data not shown).

The apparent molecular masses of S protein and its cleaved products S1 and S2 on SDS-PAGE were approximately 200, 115 and 91 kDa, respectively, obviously higher than their calculated molecular masses of 142.1, 76.7 and 64.5 kDa (Figure 2B, lanes 3–5). In addition, three S bands on the top of the glycosylated full-length S protein were detected by anti-S antisera (Figure 2B, lanes 3–5). They might represent the homodimers/homotrimers of the full-length S protein and the S1/S2 subunits, suggesting that the S1/S2 subunits may be able to form multimers after cleavage of the full-length S protein and remain associated with the virions. The possibility that these three bands may represent aggregates of several membrane-associated SARS-CoV-2 structural proteins was ruled out, as antisera against M, 3a and E did not recognize these bands (Figure 2B). Based on its reactivity to the three anti-S antisera used, the most rapidly migrating band among the three may be composed of the S2 subunits only (Figure 2B). This is consistent with the apparent molecular weight of 270 kDa for the glycosylated S2 trimers (the apparent molecular mass for the glycosylated S2 monomer was 90 kDa). The identities of these bands would be further characterized in this study.

A single band with an apparent molecular mass of 55 kDa was detected specifically with anti-N antiserum (Figure 2B, lane 6), which is higher than the calculated molecular mass of N protein (45.6 kDa) and would represent the highly phosphorylated N protein.

The calculated molecular mass of 3a is 29 kDa, however, four bands ranging with apparent molecular masses of 20–60 kDa were detected in the purified virions by anti-3a antiserum (Figure 2B, lane 7). The 50–60 kDa smear bands may represent the 3a dimers. SARS-CoV-1 dimers or tetramers were reported to form ion channels on the membrane for SARS-CoV-1 [15].

Two bands with apparent molecular masses of 26 and 23 kDa were detected in the purified virions by anti-M antiserum. They may represent the N-linked glycosylated and unglycosylated (calculated molecular mass 25 kDa) form of the M protein, respectively (Figure 2B, lane 8). The glycosylated 26 kDa M protein co-migrated with the 26-kDa 3a form on SDS-PAGE (Figure 2B, lanes 7 and 8).

When anti-virion was used, S2 and E proteins were not observed (Figure 2B, lane 10), probably as S2 located at the stalk of the S trimer and the E protein was small and only a few molecules harboured on each particle.

The relative abundance of individual viral proteins in the inactivated virions was calculated by scanning the corresponding bands in SDS-PAGE and the proportions of the S polymers, S, S1, S2, N, 3a, M and E was 8.8, 9.8, 2.7, 11.3, 57.6, 5.2, 1.6, 0.4, 0.2%, respectively (Figure 2, lane 2 and Table 1). By analysing the moler ratio, 45% of full-length S protein was uncleaved while 55% of it was cleaved.

**Table 1.** Antigenicity of viral structural proteins.

	Relative proportions of viral proteins and reactivity of antisera (%)					
Viral structural proteins	Viral proteins in virion	Total	Convalescent serum	Total	Rabbit anti-virion serum	Total
S trimer (top)	8.8	32.6	9.1		38.3	
S multimers (middle)						
S multimers (bottom)						
S full-length	9.8		9.6	32.6	7.2	53.2
S1 subunit	2.7		20.1		7.5	
S2 subunit	11.3		27.8		0.2	
N	57.6	57.6	13.6	13.6	26.3	26.3
3a dimer (2 bands)	5.2		3.8		1.5	
3a full-length	1.6		6.4		8.9	
M+3a cleaved	0.4		4.9		9.1	
M′ unglycosylated	2		4.1		0.6	
E	0.6		0.6		0.4	
Total	100		100		100	79.5

### Antigenicity of five structural proteins in virions

The relative reactivities of rabbit anti-virion and convalescent sera against the five viral proteins were detected and calculated (Figure 2B, lanes 11 and 12). It was found that the relative mass ratio of S, its multimers, subunits S1 and S2 was 32.2%, while that of the N protein was 57.6%. However, in both natural infection and animal vaccination, S protein induced higher antibody levels than did N protein (32.6% and 53.2% for S vs 13.6% and 26.3% for N). Furthermore, following immunization of inactivated virion, higher anti-S antibodies were elicited than those in natural infection (53.2% vs 32.6%). It was noted that antiserum induced by inactivated vaccine did not recognize the S2 subunit well, whereas human serum reacted strongly with both the S1 and S2 subunits. Vaccination stimulated stronger antibody responses against S polymers than natural infection. This may reflect that humoral immune response might be different between natural infection and immunization with inactivated vaccine twice [22]. It was also noticed that the 3a protein induced stronger antibody response than did the M protein.

### Homotrimerization of S, S1 and S2 on the virus particles

**Figure 3.**
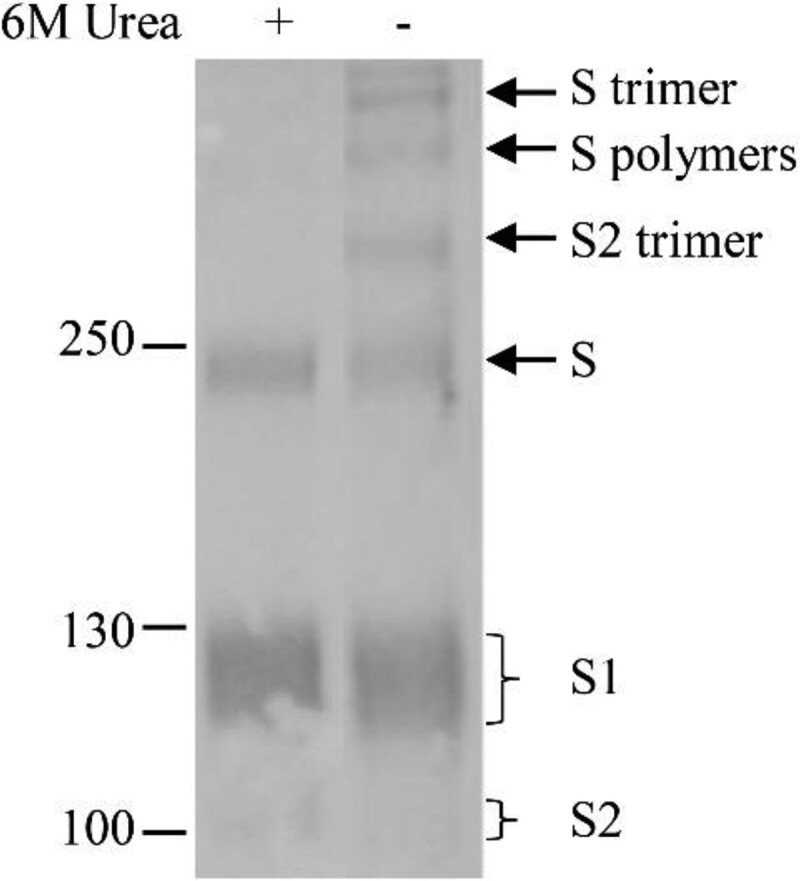
Homomultimerization of S, S1 and S2 on the virus particles. Urea (6M) was added in loading buffer containing 2% NP-40 to dissociate S multimers and proteins were separated by 8% of gradient SDS-PAGE. Rabbit α-S2 was used in WB. Molecular weight markers are indicated on the left in kilodaltons and proteins are indicated by the arrows on the right. The experiment was repeated 3 times at least.

The detection of three bands on the top of the full-length, glycosylated S suggests that they are composed of the S, and/or its cleaved S1 and S2 subunits. To confirm this, the samples were treated with strong lysis buffer containing 6 M urea, 2% NP-40, 0.2% SDS and 100 mM β-Mercaptoethanol. As shown in Figure 3, three top bands disappeared after the treatment and no extra bands were detected following treatment. The disappearance of the higher molecular mass bands on the top of the gel and the change in the ratio of S, S1 and S2 bands indicated that the S and cleaved S1 and S2 formed the trimers and/or dimers.

### Maturation of N-linked glycosylation of the S and M proteins

**Figure 4.**
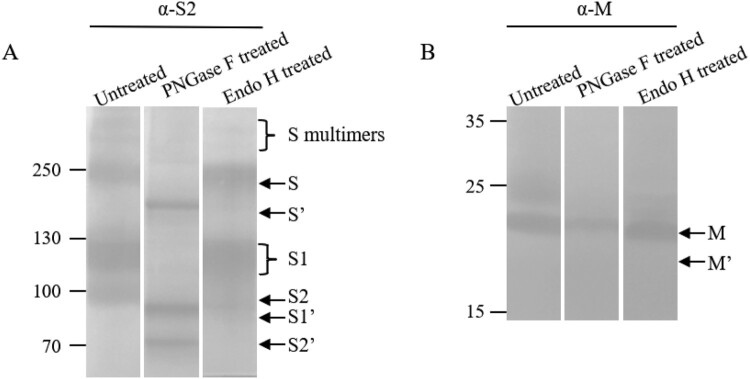
Maturation of N-linked glycosylation of the S and M proteins. Virions were untreated or treated with PNGase F or Endo H. The top and low parts of the blot were incubated with rabbit antisera α-S2 and α-M, respectively, as indicated on the top of the blots. S′, S1′, S2′ and M′ were referred to as unglycosylated forms. Molecular weight markers are indicated on the left in kilodaltons and proteins are indicated by the arrows on the right. The experiments were repeated 5 times at least.

To investigate the glycosylation of the S proteins and maturation, the purified virions were treated with PNGase F and Endo H. The full-length S and cleaved S1 and S2 bands were shifted following removal of glycans, indicative of N-linked glycosylation of these proteins as expected (Figure 4A). It was observed that the full-length S protein and S1/S2 became sharp bands after treatment with PNGase F, suggesting that the smear bands before treatment might represent multiple bands due to partial or different ways of glycosylation of the S proteins. To examine the maturation status of the glycosylated proteins, Endo H was used to treat the purified virions. It was found that the full-length S and the S1 subunit were resistant, while the S2 subunit was sensitive to the treatment (Figure 4A).

Both PNGase F and Endo H were used for treatment of the M protein (Figure 4B). The results demonstrated that the M was also an N-linked glycoprotein and was partially resistant to Endo H digestion. There were two forms of the M protein, the top one was proved to be an N-linked glycoprotein.

### Cleavage of the S proteins by Furin-like protease in Vero cells

**Figure 5.**
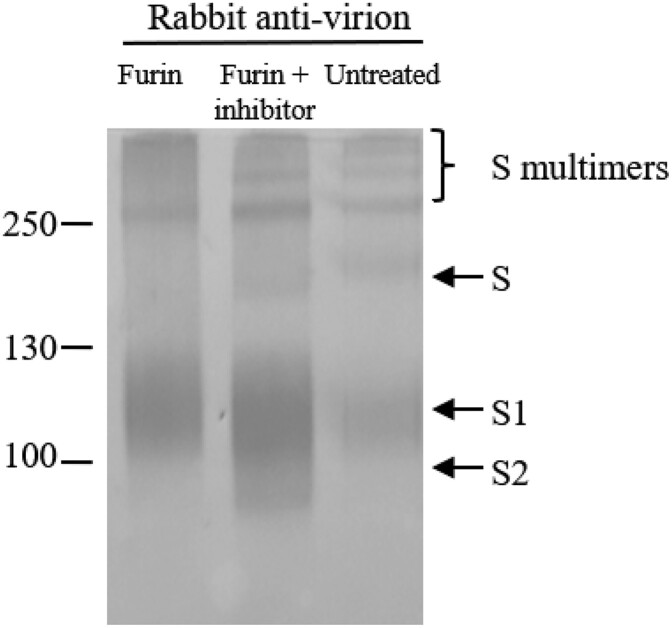
Cleavage of the S proteins by Furin-like protease in Vero cells. Viral proteins were treated with Furin or Furin + inhibitor or untreated. Proteins were separated by 4-20% gradient SDS-PAGE and transferred to Nitrocellulose membrane for WB using antiserum indicated. Molecular weight markers are indicated on the left in kilodaltons and proteins are indicated by the arrows on the right. The experiment was repeated 3 times at least.

To confirm the cleavage of the S protein by Furin-like protease in Vero cells, purified vaccine samples were treated with Furin. As shown in Figure 5, the full-length S and the top two homomultimers of S disappeared after treatment with Furin. The third multimer was resistant to Furin treatment (Figure 5), and further indicated that it was formed by S2 subunit without S1/S2 cleavage site. As the other top two multimers were sensitive to Furin treatment, they would contain the S1/S2 site. This was further confirmed by adding the furin inhibitor, band-shifts were not observed when the inhibitor was used (Figure 5). However, the cleavage at the S2′ site was not observed as the cleavage products were not identified in Vero cells when rabbit anti-virion and α-S3 serum were used (Figure 2B, lane 11, data on α-S3 not shown). Although both antisera were raised against the region between the S1/S2 and the S2′ sites, we were uncertain if they would react with the cleavage product [5]. An antibody raised against this region of the avian coronavirus infectious bronchitis virus S protein was also failed to recognize the cleavage product in the infected Vero cells [23].

### Partial or different ways of S glycosylation and the post-translational modification of viral proteins

**Figure 6.**
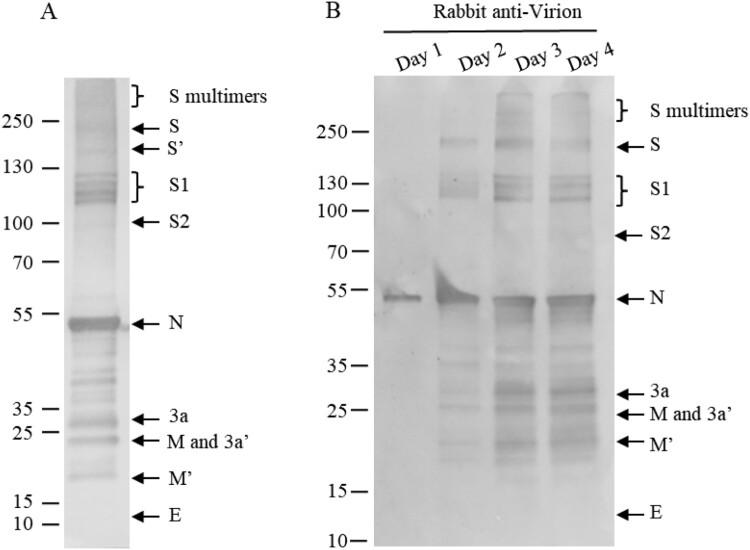
Multi-bands of S1 and time course of viral protein modifications. (A) Purified virions were analysed by 4–12% SDS-PAGE at 20mA, and transferred at 380 mA for 70 min. (B) Supernatants of infected cells were collected on days 1 to 4 post-infection and analysed by WB using rabbit-α-virion serum. The proteins were separated by 4-20% of gradient SDS-PAGE and transferred to Nitrocellulose membrane. S′ and M′, unglycosylated; 3a′ cleaved. Molecular markers are indicated on the left in kilodaltons and proteins are indicated by the arrows on the right. The experiments were repeated 3 times at least.

As the smear bands of the full-length S, S1/S2 were repeatedly observed, the purified virions were analysed in an SDS-8% PAGE at lower current. As it was shown in Figure 6A, four bands of the S1 subunit were recognized with anti-virion antiserum, confirming that the smear bands indeed represent multiple bands of the S1 subunit. It suggests that the S1subunit was probably partially N-linked-glycosylated or in different ways.

To exclude the possibility that the post-translational modifications observed were the artifacts of the purification processes in the vaccine production, the supernatants of infected Vero cells were collected on days 1 to 4 and the protein profiles were analysed by Western blotting assay (Figure 6B), showing that modifications were not artifacts of purification processes. The protein profiles from the infected cells were identical to the finished vaccine product (Figure 6A). As all these viral structural proteins, including S, 3a, M and E, were detected in supernatants of infected Vero cells, they were assembled into virions.

## Discussion

In this study, the SARS-CoV-2 S protein was confirmed to be cleaved by Furin-like protease in Vero cells and N-linked glycosylated, as the full-length and S1/S2 subunits were deglycosylated by PNGase F. These S proteins in virions were resistant to treatment of Endo H, demonstrating that they were modified and matured. It was reported that all 22 predicted N-glycosylated sites in SARS-CoV-2 S protein were used [24], whereas a report on glycosylation of SARS-CoV-1 S protein showed that among 23 potential sites, only 12 were used [25]. In this study, four different forms of S1 were identified, reflecting a complex profile of the N-linked glycosylation of SARS-CoV-2 S1 subunit in Vero cells.

Purified virions stimulated high levels of neutralizing antibody, while S fragments expressed in *E. coli* did not induce neutralizing antibody (Data not shown). The results were similar as previously reported on the immunogenicity of SARS-CoV-1. Among 5 overlapped S fragments covering the Ectodomain expressed in *E. coli,* only a C-terminal fragment including HR1 and HR2 regions stimulated neutralizing antibodies after more than 10 boosting immunizations [20]. The research on S protein of SARS also demonstrates that there are neutralizing epitopes in the S2 function domains [26].

Importantly, the Furin-like protease-cleaved S1 and S2 might form dimers and trimers, as well as the full-length S protein. The cleavage at S2′ site was not detected in infected Vero cells. It is believed that the prefusion conformation maintained if S protein is not cleaved especially at the S2′ site, and the postfusion form might induce non-neutralizing antibodies. Moderna’s mRNA-1273 vaccine candidate was designed in such a way that a transmembrane anchor and S-2P is stabilized in its prefusion conformation by two consecutive proline substitutions at amino acid positions 986 and 987 [27]. Theoretically, some epitopes in any viral antigens could induce non-neutralizing antibodies, which would potentially facilitate the entry of re-infecting virus or a closely-related virus through binding of virus-antibody complex to the Fcγ receptor on the surface of immune cells. If the post-fusion conformation of the SARS-CoV-2 S protein is proved to induce non-neutralizing and harmful antibodies, design of a vaccine candidate containing the prefusion S form would be helpful to overcome potential safety concerns. Ideally, a native form of the S protein or S subunit with dominant functional-epitopes and lack of non-neutralizing antigen epitopes is critical for the safety and efficacy, through either native selection or molecular engineering. Comparison of the antigenicity of viral structural components in this study showed that the S trimers/dimers stimulated stronger antibody response. The immunogenicity of different forms of S homotrimers/dimers needed further investigation, as understanding of the properties of these components in an inactivated vaccine may be related to its performances in the safety and efficacy in phase II and III clinical trials ongoing at the time of the writing of this report [28].

A previous study on SARS-CoV-1 3a protein showed that a truncated form was assembled into virions, while the newly synthesized 3a protein in infected cells was full-length [11]. In this study, SARS-CoV-2 3a protein was shown to be presented in two forms in virions as well as 3a dimers. It is not clear whether the more rapidly migrating 3a form is a premature-terminated product or post-translationally cleaved form. The 3a protein was shown to stimulate humoral immune response as strong as the M protein, despite more abundancy of M protein in the virions. SARS-CoV-1 3a protein was reported to be located on the surface of Golgi apparatus, associated with apoptosis, ion channel formation on cell membrane and enhancement of virus release from infected cells [14,15,29]. Further studies would be required to explore if the presence of anti-3a antibodies in a portion, at least, of patients is related to the recovery or severity of the disease, and if humoral immune response against 3a protein induced by an inactivated vaccine would be beneficial.

Most coronavirus M proteins were N-linked glycosylated, including SARS-CoV-1 M protein that contains a single N-linked glycosylation site[29,30]. In infected cells and purified virions, SARS-CoV-1 M protein remained Endo H sensitive, suggesting incomplete maturation of the protein [31]. This is consistent with the data on SARS-CoV-2 M protein in this study. Also, a previous study on mouse hepatitis virus (MHV) reported that a monoclonal antibody against the M protein showed *in vitro* neutralizing activity, protecting mice from lethal virus challenge [8]. It is worthy to investigating whether anti-M antibodies have the neutralizing activity against SARS-CoV-2.

The profiles of the characterized viral proteins and their post-translational modifications presented in this study were derived from SARS-CoV-2 virions of inactivated, purified products through many manufactural processes. We cannot totally rule out the possibility that some of the modifications might be artifacts caused by the purification processes and cellular enzymes during cell lysis. Comparative studies and confirmation of the post-translational modifications of these proteins would be needed in virions purified through other well-designed procedures or directly from infected human cells in patients. Cleavage of Furin-like proteases should be confirmed by transient expression of S in Vero and other cells. On the other hand, as these pieces of information are derived from the end-products of an actual vaccine candidate, the elucidation of these modifications and their effects on the immunogenicity, safety and efficacy of the vaccine products would be useful and important. For example, it would provide guidelines on how to design a vaccine candidate which mimics the native, immunogenicity-competent form of the S subunit vaccine candidate.

Different platforms of vaccines against COVID-19 have their distinct advantages and disadvantages. For an inactivated and whole virus vaccine, this study provides evidence that native conformation of S homotrimers/dimers remains in inactivated virions and contains complete neutralizing epitopes of different functional domains. As a mature technology platform, inactivated viral vaccines would be readily achievable once the main hurdles of virus isolation and low yields in culture cells are overcome. However, as some of the structural components and their post-translational modifications in the whole inactivated virions may be immunopathological, detailed studies of the structure, conformation immunogenicity and functions of these proteins are required for the generation of safe and efficacious vaccines against this disease [32] . Data presented in this study would have set a starting point for such efforts.
